# Iatrogenic and spontaneous preterm birth in England: A population‐based cohort study

**DOI:** 10.1111/1471-0528.17291

**Published:** 2022-10-03

**Authors:** Harriet Aughey, Jennifer Jardine, Hannah Knight, Ipek Gurol‐Urganci, Kate Walker, Tina Harris, Jan van der Meulen, Jane Hawdon, Dharmintra Pasupathy, the NMPA Project Team, Andrea Blotkamp, Fran Carroll, Megan Coe, George Dunn, Rebecca Geary, Alissa Harvey, Emma Heighway, Asma Khalil, Julia Langham, Lindsey Mamza, Patrick Muller, Natalie Moitt, Sophie Relph, Louise Thomas, Lara Waite, Kirstin Webster

**Affiliations:** ^1^ Royal College of Obstetricians and Gynaecologists London UK; ^2^ University Hospitals Bristol NHS Foundation Trust Bristol UK; ^3^ Department of Health Services Research and Policy London School of Hygiene and Tropical Medicine London UK; ^4^ Faculty of Health and Life Sciences De Montfort University Leicester UK; ^5^ Royal Free NHS Foundation Trust London UK; ^6^ Reproduction and Perinatal Centre, Faculty of Medicine and Health University of Sydney Sydney New South Wales Australia

**Keywords:** iatrogenic, induction, preterm birth, spontaneous

## Abstract

**Objective:**

To describe the rates of and risk factors associated with iatrogenic and spontaneous preterm birth and the variation in rates between hospitals.

**Design:**

Cohort study using electronic health records.

**Setting:**

English National Health Service.

**Population:**

Singleton births between 1 April 2015 and 31 March 2017.

**Methods:**

Multivariable Poisson regression models were used to estimate adjusted risk ratios (adjRR) to measure association with maternal demographic and clinical risk factors.

**Main outcome measures:**

Preterm births (<37 weeks of gestation) were defined as iatrogenic or spontaneous according to mode of onset of labour.

**Results:**

Of the births, 6.1% were preterm and of these, 52.8% were iatrogenic. The proportion of preterm births that were iatrogenic increased after 32 weeks. Both sub‐groups were associated with previous preterm birth, extremes of maternal age, socio‐economic deprivation and smoking. Iatrogenic preterm birth was associated with higher body mass index (BMI) (BMI >40 kg/m^2^ adjRR 1.59, 95% CI 1.50–1.69) and previous caesarean (adjRR 1.88, 95% CI 1.83–1.95). Spontaneous preterm birth was less common in women with a higher BMI (BMI >40 kg/m^2^ adjRR 0.77, 95% CI 0.70–0.84) and in women with a previous caesarean (adjRR 0.87, 95% CI 0.83–0.90). More variation between NHS hospital trusts was observed in rates of iatrogenic, compared with spontaneous, preterm births.

**Conclusions:**

Just over half of all preterm births resulted from iatrogenic intervention. Iatrogenic births have overlapping but different patterns of maternal demographic and clinical risk factors to spontaneous preterm births. Iatrogenic and spontaneous sub‐groups should therefore be measured and monitored separately, as well as in aggregate, to facilitate different prevention strategies. This is feasible using routinely acquired hospital data.

## Abstract 2

This article includes Author Insights, a video abstract available at: https://vimeo.com/745289966.

## Graphical abstract

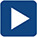

## 1 INTRODUCTION

Preterm birth is the single largest cause of neonatal morbidity and mortality in many countries.^1^, ^2^, ^3^, ^4^ Being born preterm confers an increased lifelong risk of disability and chronic disease.^2^, ^3^ The costs of preterm birth are high, inversely related to gestation at birth, and persist throughout childhood.^4^, ^5^ Preterm birth is also associated with substantial impacts upon family life.^4^ Prevention of preterm birth is, therefore, an important aim in modern obstetric practice.^6^, ^7^

Measurement of the rate of preterm birth and comparison between providers is desirable to evaluate interventions that aim to reduce preterm birth^8^ and enable clinical benchmarking. However, the aetiology of preterm birth is complex and treating it as a single outcome may hinder appropriately targeted interventions.

It has been recognised that it is important to distinguish between spontaneous and iatrogenic birth,^9^ depending on whether birth is initiated by the provider of maternity care. Iatrogenic preterm birth is indicated in response to maternal illness or signs of fetal compromise; these are increasingly detected and acted on, with benefits to maternal health in some situations.^10^ Unsurprisingly, therefore, iatrogenic preterm birth is increasing in many high‐income countries^11^, ^12^, ^13^, ^14^, ^15^, ^16^ potentially limiting progress towards reducing overall rates of preterm birth.^12^, ^17^, ^18^

In the UK, overall rates of preterm birth have remained relatively static in recent years. It is not known how the rates of iatrogenic and spontaneous preterm birth have changed within this aggregated total.^1^ Using a large, routinely collected data set, this study describes the rates of iatrogenic and spontaneous preterm birth in England; the maternal demographic and clinical risk factors associated with each group; the recorded maternal and fetal indications for iatrogenic preterm birth and the variation in preterm birth rates between hospital trusts. By describing these two component outcomes of preterm birth separately, we demonstrate that these can be regarded as separate outcomes to benchmark local and national performance.

## 2 METHODS

The data for this study were obtained from two population‐level electronic data sets, linked together for the purposes of a national audit of maternity care, including births that occurred from 1 April 2015 to 31 March 2017.^19^ Data from maternity information systems in hospitals providing maternity services in the English National Health Services (NHS) were linked to data from the Hospital Episode Statistics (HES), the database that collects administrative data for admissions to NHS hospitals. The linkage process was based on the mother's and baby's date of birth and the mother's NHS number, as previously described.^20^

The maternity information system record contained information about gestational age, the mode of onset of labour, the birth, and maternal and neonatal characteristics including parity, ethnic group and gestational age. Socio‐economic status was evaluated using the Index of Multiple Deprivation, an area‐level measure of deprivation identified by the woman's recorded postcode in maternity information systems.^21^ Information about maternal diagnoses including pre‐eclampsia and gestational diabetes was available in HES. Women's previous birth record, including the mode of birth and previous preterm birth, was available using a ‘look‐back’ approach in HES where all previous records for the woman since 2000 in English NHS hospitals were considered.^22^, ^23^

All 1 254 484 live births of at least 22 weeks of gestation in 133 NHS hospital trusts were eligible for inclusion. Births in 23 hospital trusts were excluded from the study because of poor quality data (<70% of records with complete information on all of: stillbirth or livebirth; gestational age; method of labour onset; and delivery method), or poor linkage (<70% of records with complete identifiers to enable linkage to HES). Stillbirths were excluded because it was not possible to identify whether a stillbirth occurred antepartum or intrapartum. Multiple births were excluded because the frequency of preterm birth in multiple pregnancies differs and therefore should be considered separately. In all, 9283 births without a record of labour onset were excluded, representing less than 1% of the cohort. In total, 963 800 (93.1% of births in included hospitals) live singleton births with complete data about gestational age, method of labour onset, and delivery method were included in the analyses (Figure S1).

Births were considered preterm if they had a recorded gestation of 37 completed weeks or fewer at birth. Births were defined as iatrogenic if there was a record of induction of labour or of caesarean section (CS) before the onset of labour and as spontaneous if the recorded onset was spontaneous. Details of variable definitions are available in Table S1.

To investigate associations between risk factors and outcomes, chi‐square tests were used. Multivariable Poisson regression models with robust standard errors were used to estimate the association between maternal risk factors for preterm birth overall and stratified into iatrogenic and spontaneous. The purpose of these models was to describe the prognostic factors for each of spontaneous and iatrogenic preterm birth. The maternal risk factors included were age, body mass index (BMI), ethnicity, socio‐economic status, smoking status at booking, parity, previous CS and previous preterm birth. Interaction terms were included for parity and each of previous CS and previous preterm birth. In estimating the strength of the relationship between each risk factor and preterm birth in regression analyses, clustering of preterm births in individual units was accounted for by using robust standard errors, which do not change the estimate of the association but widen the confidence intervals to allow for similarities between individuals within clusters.^24^ For regression analyses, missing values were imputed using multiple imputation by chained equations with statistical coefficients obtained using ten imputed data sets, pooled using Rubin's rules.^25^ The imputation model included all variables in the analysis model.

Funnel plots were used to visually explore the variation between hospital organisations in rates of preterm birth, both overall and disaggregated into iatrogenic and spontaneous. In these plots, results for each hospital organisation were adjusted using the multivariable Poisson regression models described above. These plots indicate whether the difference between rate of preterm birth in a hospital organisation and the national mean is statistically significant at the 5% level (if the preterm birth rate is outside the inner funnel limit) or 0.2% level (if the preterm birth rate is outside the outer funnel limit), given the different case‐mix of women treated in each individual hospital.^26^

Two clinicians (JJ and DP) mapped possible indications for iatrogenic preterm birth to International Classification of Diseases 10th revision codes. These were then identified in the maternal record. Further details of codes used are available in Table S2.

Three sensitivity analyses were conducted to assess whether the effects of maternal risk factors were sensitive to the inclusion criteria and methods used. In the first, births associated with preterm prelabour rupture of membranes (PPROM) were excluded. PPROM represents an area of relative uncertainty as it may represent the beginning of a mechanistic process leading to spontaneous preterm birth. It may also result in iatrogenic preterm birth due to clinical need such as infection. Excluding PPROM therefore allows a more robust evaluation of factors related to ‘pure’ spontaneous and iatrogenic preterm birth. In the second sensitivity analysis, maternal diabetes, hypertension and pre‐eclampsia/eclampsia were included in the regression models to explore whether the observed associations were explained by these maternal risk factors. Further details are available in Table S2. Finally, we tested whether our results were robust to alternative methods of handling missing data by repeating the regression analysis in the subset of records with complete information about all covariates. STATA v14.1 (StataCorp, College Station, TX, USA) was used for all analyses.

## 3 RESULTS

**FIGURE 1:**
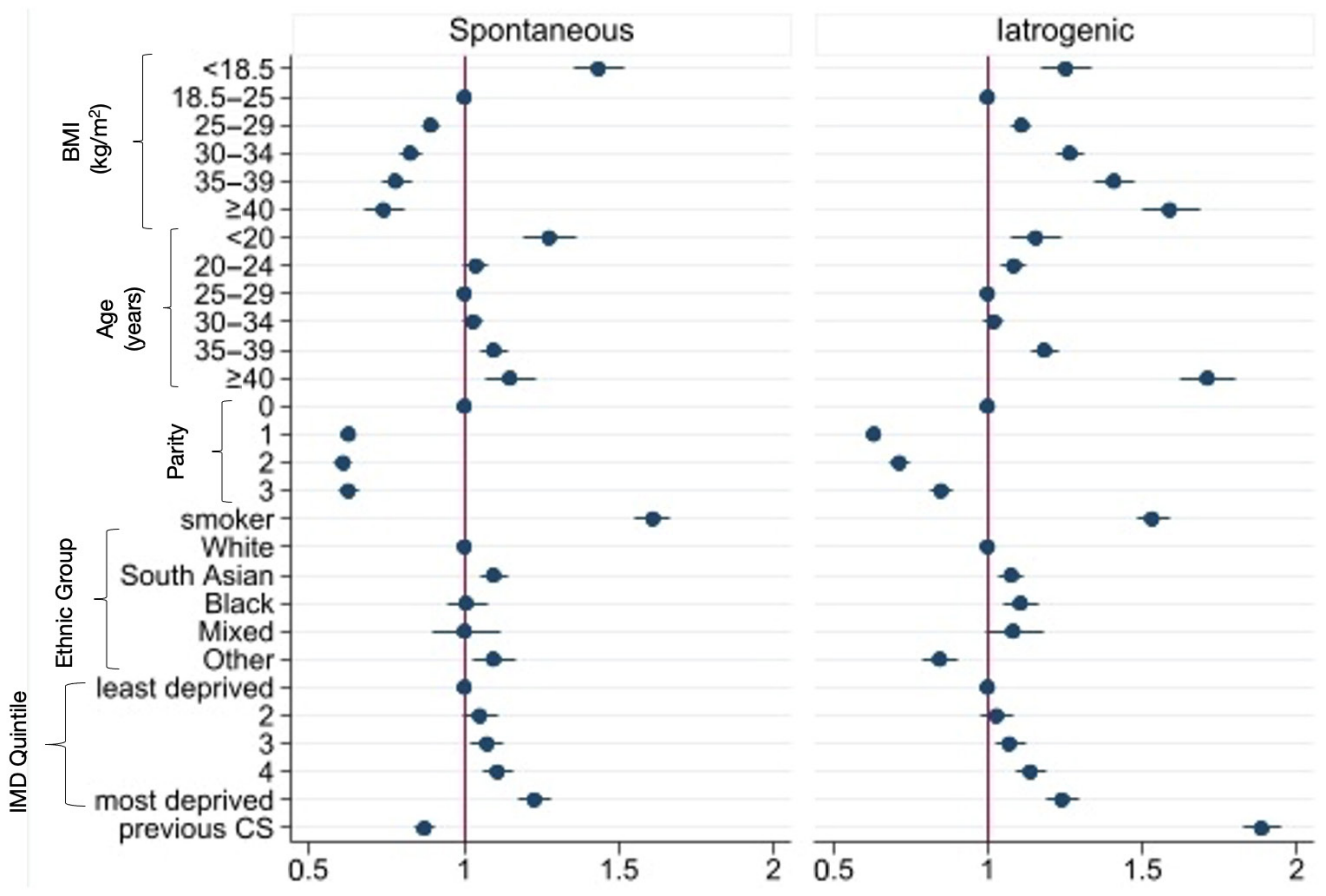
Risk‐adjusted patterns of association for spontaneous and iatrogenic preterm birth among 963 800 women who gave birth in England between 1 April 2015 and 31 March 2017. Note that the relationship with previous preterm birth is outwith the axis because of the strength of association (spontaneous preterm birth adjusted risk ratio [adjRR] 6.53, 95% CI 6.32–6.75; iatrogenic preterm birth adjRR 3.34, 95% CI 3.22–3.46).

The cohort included 963 800 women who had a singleton live birth in England between 1 April 2015 and 31 March 2017 (Figure S1). A total of 58 850 babies (6.1%) were born preterm (Table 1), of which 31 097 (52.8%) births were iatrogenic in onset. Spontaneous preterm birth was more prevalent in the early preterm period and iatrogenic births comprised a larger proportion of late preterm births (Figure 1).

**TABLE 1:** Characteristics of 963 800 women who had singleton live births in England between 1 April 2015 and 31 March 2017, and rate of spontaneous and iatrogenic preterm birth among women with each characteristic

Characteristic	Number of women with characteristic (%)	Proportion of women with each characteristic who had a preterm birth, *n* (%)		
		Preterm birth overall	Spontaneous preterm birth	Iatrogenic preterm birth
All women	963 800	58 850 (6.1)	27 753 (2.9)	31 097 (3.2)
Maternal age (years)				
<20	31 244 (3.3)	8.2	4.6	3.7
20–24	145 091 (15.1)	6.6	3.2	3.4
25–30	273 872 (28.5)	5.8	2.8	3.0
30–34	301 956 (31.5)	5.7	2.7	2.9
35–39	168 586 (17.6)	6.2	2.7	3.5
40+	38 744 (4.0)	8.0	2.8	5.2
Missing	4307 (0.5)	7.0	2.9	4.1
Maternal BMI (kg/m^2^)				
<18.5	24 451 (3.0)	8.4	4.8	3.6
18–24.9	391 957 (47.4)	5.6	2.9	2.7
25–29.9	234 607 (28.4)	5.7	2.6	3.1
30–34.9	107 898 (13.1)	6.2	2.4	3.7
35–39.9	44 428 (5.4)	6.6	2.3	4.3
40+	22 807 (2.8)	7.2	2.2	5.0
Missing	137 652 (14.3)	7.6	3.6	3.9
Ethnic group				
White	667 327 (76.2)	6.1	2.9	3.2
South Asian	112 037 (12.8)	6.5	3.0	3.5
Black	42 351 (4.8)	6.7	2.6	4.1
Mixed	15 595 (1.8)	6.5	2.9	3.5
Other	39 004 (4.5)	5.4	2.9	2.6
Missing	87 486 (9.1)	5.7	2.9	2.8
Socio‐economic deprivation quintile^a^				
Least deprived	150 773 (16.5)	5.0	2.4	2.6
2	125 401 (13.7)	5.4	2.6	2.8
3	170 630 (18.6)	5.7	2.7	3.0
4	206 181 (22.5)	6.3	2.9	3.4
Most deprived	263 309 (28.7)	7.3	3.3	3.9
Missing	47 506 (4.9)	6.0	3.1	2.9
Smoking status at booking				
Non‐smoker	693 388 (86.6)	5.5	2.5	2.9
Smoker	107 337 (13.4)	9.4	4.6	4.9
Unknown	163 075 (16.9)	6.7	3.3	3.4
Parity				
0	407 989 (42.3)	6.2	3.2	3.1
1	346 627 (36.0)	5.2	2.4	2.8
2	126 256 (13.1)	6.4	2.8	3.6
3+	82 928 (8.6)	8.8	3.6	5.2
Previous CS				
Previous CS	133 907 (14.0)	8.3	2.7	5.6
Missing	4157 (0.5)	6.1	2.8	3.3
Previous PTB	41 762 (4.3)	23.7	12.8	10.9
Pre‐existing hypertension	5339 (0.6)	15.7	2.7	13.0
Diabetes (pre‐existing or gestational)	57 714 (6.0)	10.9	3.5	7.4
Pre‐eclampsia or eclampsia	17 916 (1.9)	27.8	2.1	25.7

Abbreviations: CS, caesarean section; PTB, preterm birth.

^a^ Socio‐economic deprivation is reported in quintiles where 1 is the least deprived and 5 is the most deprived.

The highest rates of preterm birth were seen in women with a previous preterm birth; 12.8% of these women had spontaneous and 10.9% had iatrogenic preterm births (Table 1). Following adjustment for demographic and clinical risk factors, there remained a substantial association between both sub‐groups of preterm birth and previous preterm birth; the relationship was stronger for spontaneous than iatrogenic preterm birth (iatrogenic adjusted risk ratio [adjRR] 3.34, 95% CI 3.22–3.46; spontaneous adjRR 6.53, 95% CI 6.32–6.75).

Increasing socio‐economic deprivation was associated with increasing risk of both sub‐groups of preterm birth. Women in the most deprived neighbourhoods were approximately 20% more likely to have either a spontaneous (adjRR 1.21, 95% CI 1.16–1.26) or iatrogenic (adjRR 1.24, 95% CI 1.19–1.29) preterm birth compared with women in the least deprived neighbourhoods. Similarly, smoking was associated with an increased risk in both sub‐groups (spontaneous, adjRR 1.61, 95% CI 1.56–1.67); iatrogenic (adjRR 1.55, 95% CI 1.50–1.60) (Table 2, Figure 1).

**TABLE 2:** Characteristics of women having a preterm birth (22^+0^ to 36^+6^ weeks of gestation), disaggregated into spontaneous and iatrogenic births, compared with those having a term birth, among 963 800 singleton live births in England in 2015–17

Characteristics	Spontaneous preterm birth		Iatrogenic preterm birth	
	Crude risk ratio (95% CI)^a^	Adjusted risk ratio (95% CI)^b^	Crude risk ratio (95% CI)^a^	Adjusted risk ratio (95% CI)^b^
Maternal age (years)				
<20	1.63 (1.54–1.72)	1.32 (1.24–1.39)	1.23 (1.15–1.30)	1.16 (1.08–1.24)
20–24	1.16 (1.12–1.20)	1.05 (1.01–1.08)	1.12 (1.08–1.16)	1.09 (1.04–1.13)
25–30	Ref	Ref	Ref	Ref
30–34	0.98 (0.95–1.01)	1.05 (1.02–1.08)	0.98 (0.95–1.01)	1.02 (0.99–1.05)
35–39	0.97 (0.93–1.00)	1.09 (1.04–1.13)	1.16 (1.13–1.20)	1.18 (1.14–1.23)
40+	1.01 (0.95–1.08)	1.17 (1.10–1.25)	1.74 (1.66–1.82)	1.71 (1.62–1.80)
Maternal BMI (kg/m^2^)				
<18.5	1.66 (1.56–1.76)	1.43 (1.35–1.52)	1.37 (1.28–1.47)	1.25 (1.17–1.34)
18.5–24.9	Ref	Ref	Ref	Ref
25–29.9	0.89 (0.86–0.92)	0.90 (0.87–0.93)	1.16 (1.12–1.19)	1.11 (1.08–1.14)
30–34.9	0.84 (0.81–0.88)	0.84 (0.80–0.87)	1.39 (1.34–1.44)	1.27 (1.22–1.31)
35–39.9	0.82 (0.77–0.87)	0.79 (0.75–0.84)	1.60 (1.53–1.68)	1.41 (1.34–1.48)
40+	0.79 (0.72–0.86)	0.77 (0.70–0.84)	1.87 (1.76–1.98)	1.59 (1.50–1.69)
Ethnic group				
White	Ref	Ref	Ref	Ref
South Asian	1.04 (1.01–1.08)	1.08 (1.04–1.12)	1.10 (1.06–1.13)	1.08 (1.04–1.12)
Black	0.92 (0.87–0.97)	1.00 (0.94–1.06)	1.27 (1.21–1.33)	1.11 (1.05–1.16)
Mixed	1.03 (0.93–1.13)	1.00 (0.91–1.10)	1.10 (1.01–1.19)	1.05 (0.97–1.14)
Other	0.99 (0.93–1.05)	1.08 (1.01–1.14)	0.80 (0.75–0.85)	0.86 (0.80–0.91)
Socio‐economic deprivation quintile				
1 (least deprived)	Ref	Ref	Ref	Ref
2	1.07 (1.02–1.12)	1.04 (1.00–1.09)	1.05 (1.01–1.10)	1.03 (0.98–1.08)
3	1.11 (1.06–1.16)	1.06 (1.01–1.11)	1.14 (1.09–1.19)	1.07 (1.02–1.12)
4	1.21 (1.16–1.26)	1.11 (1.06–1.15)	1.27 (1.22–1.32)	1.14 (1.09–1.19)
5 (most deprived)	1.38 (1.33–1.44)	1.21 (1.16–1.26)	1.48 (1.43–1.54)	1.24 (1.19–1.29)
Smoking status				
Non‐smoker	Ref	Ref	Ref	Ref
Smoker	1.78 (1.73–1.84)	1.61 (1.56–1.67)	1.68 (1.63–1.73)	1.53 (1.48–1.59)
Parity				
0	Ref	Ref	Ref	Ref
1	0.77 (0.75–0.80)	0.63 (0.61–0.64)	0.89 (0.87–0.92)	0.63 (0.61–0.65)
2	0.87 (0.84–0.91)	0.60 (0.58–0.63)	1.17 (1.13–1.21)	0.70 (0.68–0.73)
3+	1.14 (1.10–1.19)	0.63 (0.60–0.66)	1.70 (1.64–1.75)	0.83 (0.79–0.86)
Previous CS	0.93 (0.90–0.96)	0.87 (0.83–0.90)	1.97 (1.92–2.02)	1.88 (1.83–1.95)
Previous PTB	5.27 (5.12–5.42)	6.53 (6.32–6.75)	3.77 (3.66–3.89)	3.34 (3.22–3.46)

Abbreviations: CS, caesarean section; PTB, preterm birth; Ref, reference category.

^a^ Risk ratio compared with the reference category.

^b^ Compared with reference category, adjusted for listed factors. *p* < 0.001 for all associations.

Both iatrogenic and spontaneous preterm births were more common at the extremes of maternal age (Table 2). Women aged under 20 years were at higher risk of either a spontaneous (adjRR 1.32, 95% CI 1.24–1.39) or iatrogenic (adjRR 1.16, 95% CI 1.08–1.24) preterm birth when compared with women aged 25–30 years. A similar pattern was observed for women aged over 40 years (spontaneous adjRR 1.17, 95% CI 1.10–1.25, iatrogenic adjRR 1.71, 95% CI 1.62–1.80).

Opposing directions of association were found for other characteristics. Elevated BMI was associated with higher rates of iatrogenic preterm birth (adjRR for BMI >40 kg/m^2^, 1.57, 95% CI 1.48–1.67), but lower rates of spontaneous term birth (adjRR for BMI >40, 0.77, 95% CI 0.70–0.84) (Table 2, Figure 1). Previous CS was associated with an almost doubling of the rate of iatrogenic preterm birth (adjRR 1.88, 95% CI 1.83–1.95) but a reduction in the rate of spontaneous preterm birth (adjRR 0.87, 95% CI 0.83–0.90).

South Asian women were approximately 8% more likely to have either a spontaneous (adjRR 1.08, 95% CI 1.04–1.12) or iatrogenic (adjRR 1.08, 95% CI 1.04–1.12) preterm birth when compared with white women. Black women had similar rates of spontaneous (adjRR 1.00, 95% CI 0.94–1.06) but higher rates of iatrogenic (adjRR 1.11, 95% CI 1.05–1.16) preterm birth.

Our findings were not materially different in the sensitivity analyses excluding 3842 preterm births with PPROM (14.4% of all preterm births) and restricted to complete cases (Tables S5 and S6).

Increased rates of iatrogenic, but not spontaneous, preterm birth were seen in women with pre‐existing hypertension (13.0%), diabetes (7.4%) or pre‐eclampsia/eclampsia (25.7%) (Table 1). In the sensitivity analysis that included adjustment for maternal hypertensive and diabetic disorders these conditions were strongly associated with iatrogenic preterm birth (adjRR for pre‐eclampsia or eclampsia, 8.34, 95% CI 8.07–8.62; adjRR for pre‐existing or gestational diabetes, 2.16, 95% CI 2.01–2.31). In this analysis, the association between iatrogenic preterm birth and socio‐economic deprivation remained, the association with raised BMI was attenuated but remained (BMI of 40 kg/m^2^ or higher compared with BMI 18.5–24.9 kg/m^2^; adjRR 1.13, 95% CI 1.05–1.21) and the associations between preterm birth and maternal South Asian and black ethnicity were no longer present (Table S7).

### 3.1 Fetal and maternal indications for iatrogenic preterm birth

A total of 88.4% of iatrogenic preterm births had a potential indication for delivery recorded in HES. The most common fetal indications were suspected distress (26.8%) and growth restriction (22.9%); the most common maternal indications were hypertensive disease (18.2%) and diabetes (12.4%) (Table S4)

### 3.2 Variation between hospital trusts

**FIGURE 2:**
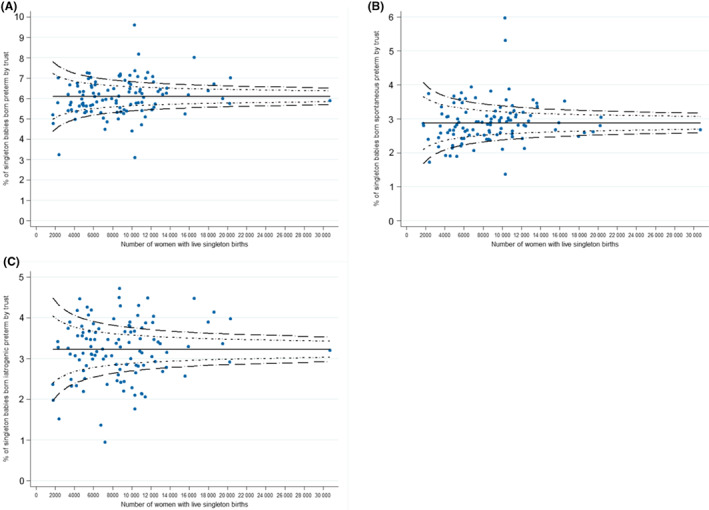
Funnel plots showing the risk‐adjusted proportion of singleton preterm births by trust of birth: (A) total preterm birth, (B) spontaneous preterm birth and (C) iatrogenic preterm birth.

Risk‐adjusted rates of iatrogenic preterm birth ranged from 0.95% to 4.72% (interquartile range 2.78%–3.73%) and of spontaneous preterm birth from 1.37% to 5.96% (interquartile range 2.55%–3.12%) in the 110 hospital trusts included in this study. The funnel plots demonstrate larger between‐hospital variation in the rates of iatrogenic preterm births than rates of spontaneous preterm births (Figure 2).

## 4 DISCUSSION

### 4.1 Main findings

Preterm birth accounted for 6.1% of all singleton live births in England in 2015–17; just over half (52.8%) were born as a result of iatrogenic intervention. This figure is larger than the 25–30% previously quoted for the UK^1^, ^27^ and is consistent with reported increases in iatrogenic preterm birth globally, particularly in high‐income settings.^7^, ^28^ Much of the observed variation seen in preterm birth rates is accounted for by iatrogenic preterm birth.

Iatrogenic and spontaneous preterm birth have areas of overlap in their risk factors. Both are strongly associated with previous preterm birth, socio‐economic deprivation and smoking. However, there are also important differences between the two sub‐groups. Older women, those with higher BMI, previous CS and medical comorbidities are more likely to experience iatrogenic preterm birth. Spontaneous preterm birth is more common among younger women, but less common in those with a higher BMI and who have had a previous CS; this may be partially accounted for by these women being more likely to have an iatrogenic intervention if they show signs of labour.

The observed association between ethnic group and iatrogenic preterm birth disappeared after adjustment for maternal hypertension, pre‐eclampsia and diabetes, suggesting that differences in iatrogenic preterm birth between ethnic groups is largely accounted for by different prevalence of comorbidities in non‐white ethnic groups. Similarly, we show that the association between iatrogenic preterm birth and obesity is partly explained by the higher prevalence of maternal medical conditions among women with a raised BMI. In contrast, the association between socio‐economic deprivation and preterm birth remained consistent across all analyses.

### 4.2 Strengths and limitations

This study uses a large contemporary data set^19^ containing rich information about maternal risk factors and neonatal characteristics. Approximately 92% of births that occurred in England in the time period were included. The main reason for exclusion was poor completeness (<70%) of records at the hospital trust level; this led to 23 (17%) hospital trusts being excluded from the study. Excluding trusts with poor‐quality data, rather than exclusion of individual patient data, minimises the risk of systematic bias.

The adjusted results may be affected by residual confounding from information not available to us. For example, we were unable to include previous early pregnancy loss or cervical surgery, which are both recognised risk factors for preterm birth.^18^, ^29^ Furthermore, our data were not sufficiently granular to identify the stage of labour at which a previous CS had occurred. A minority of CS occur at full dilatation and it has been observed elsewhere that this history is associated with subsequent spontaneous preterm birth; this association is masked in our study by the majority of CS which occur prelabour or in early labour and are not associated with an increase in spontaneous preterm birth.

The absence of some risk factors for preterm birth from our data set may also account for some of the observed variation in iatrogenic preterm birth. For example, we could not account for neonatal conditions requiring surgery, for which iatrogenic birth may be planned in specific units necessitating in utero transfer. Such births, however, make up a small proportion of all preterm births.

Our categorisation of preterm birth does not separately consider PPROM. The approach to PPROM in the literature is heterogeneous, with some studies treating it as a distinct category^9^, ^11^ and others including it as spontaneous.^18^ A sensitivity analysis excluding women with PPROM from this analysis did not reveal substantial differences in the results.

### 4.3 Implications (in light of other evidence)

This study shows that the proportion of preterm births in England that are iatrogenic in onset is greater than has previously been recognised^1^, ^27^ occurring more frequently than spontaneous preterm births. This may be partially attributable to changes in risk factor profile over time, in particular increasing maternal age and the increasing prevalence of maternal obesity,^20^, ^30^ both of which are associated with increased risk of iatrogenic preterm birth. Changes in obstetric practice following international initiatives to reduce stillbirth may have contributed to an increase in iatrogenic preterm birth over time.^10^, ^31^

Both spontaneous and iatrogenic preterm birth are strongly associated with smoking and therefore antenatal interventions to encourage smoking cessation^8^ should be prioritised.^32^, ^33^ Understanding and addressing the association between preterm birth and socio‐economic deprivation is complex and will require primary care, public health and broader societal policy interventions.

For iatrogenic preterm birth, prevention may be targeted towards modifiable upstream factors to ensure that women enter pregnancy well, with a normal BMI, as well as appropriate and timely surveillance of comorbidities and pregnancy complications. This requires multiagency involvement extending beyond obstetric and midwifery care. Research into complications of pregnancy, such as pre‐eclampsia and diabetes must focus on identifying pregnancies for which it is possible to delay birth while ensuring optimal maternal outcomes.

For spontaneous preterm birth, targeted monitoring and intervention for women identified at higher risk is effective.^6^, ^27^ The identification of women at risk of preterm birth is beneficial even where primary prevention is not feasible, as this allows for optimal perinatal and neonatal management, thereby improving neonatal outcomes.^34^

The study demonstrates the feasibility of using routinely acquired hospital administrative data to measure risk‐adjusted rates of iatrogenic and spontaneous preterm birth and to compare these between hospitals. This offers potential value as a tool for measuring progress towards reducing preterm birth. The primary goal of iatrogenic preterm birth is to improve outcomes for the mother and baby; avoiding or delaying iatrogenic preterm birth may be associated with poorer outcomes.^10^ Nevertheless, preterm birth, be it iatrogenic or spontaneous, confers risks of significant sequelae.^2^, ^3^ The ‘optimal’ rate of iatrogenic preterm birth is not clear, however, variation in rates of iatrogenic preterm birth seen between NHS trusts (Figure 2) indicates variation in practice and therefore presents an opportunity for benchmarking to improve performance,^26^ not only to reduce overall preterm birth but also to reduce, where appropriate, preventable maternal and neonatal morbidity.

## 5 CONCLUSIONS

This study adds to the literature supporting the finding that iatrogenic and spontaneous preterm birth are associated with different patterns of maternal demographic and clinical risk factors.^35^ Measuring spontaneous and iatrogenic preterm birth separately as well as in aggregate will facilitate accurate evaluation of interventions aimed at preventing preterm birth, a better understanding of the impact of changes in maternity policy on preterm birth rates, and more targeted identification of areas for intervention within the two sub‐groups. Public health measures to decrease smoking, mitigate for ethnic and socio‐economic inequality, and reduce maternal weight at the onset of pregnancy are necessary to reduce preterm birth.

## AUTHOR CONTRIBUTIONS

DP conceived the study. All authors contributed to the design of the study. JJ performed the analyses. HA reviewed the literature. HA and JJ wrote the manuscript. All authors critically revised the manuscript for important intellectual content and have given approval of the final version before submission.

## FUNDING INFORMATION

The National Maternity and Perinatal Audit is commissioned by the Healthcare Quality Improvement Partnership (HQIP) as part of the National Clinical Audit and Patient Outcomes Programme and funded by NHS England and the Scottish and Welsh Governments. Neither HQIP nor the funders had any involvement in designing the study; collecting, analysing and interpreting the data; writing the report; or in making the decision to submit the article for publication.

## CONFLICT OF INTERESTS

The authors declare no conflict of interests. HA, JJ, HK, JH, TH, IGU, DP have all received support to deliver the National Maternity and Perinatal Audit. JvdM chairs the National Maternity and Completed disclosure of interests form available to view online as supporting information.

## ETHICS APPROVAL

This study used data collected to evaluate service provision and performance and therefore it was exempt from ethical review by the NHS Health Research Authority. The use of data without patient consent was approved by the Confidentiality Advisory Group of the NHS Health Research Authority for the purpose of national clinical audit and health service evaluation (16/CAG/0058).

## Supporting information


Appendix S1

ICMJE

ICMJE

ICMJE

ICMJE

ICMJE

ICMJE

ICMJE

ICMJE

ICMJE

## Data Availability

The data are available for further research and service evaluation following approval from the data controllers, which are the Healthcare Quality Improvement Partnership (HQIP; www.hqip.org.uk) for the data derived from the maternity information systems and NHS Digital for Hospital Episode Statistics.

## References

[1] National Institute for Health and Care Excellence . Preterm labour and birth [Internet]. 2015 [cited 2022 Jan 10]. Available from: www.nice.org.uk/guidance/ng25 31971700

[2] Mangham LJ , Petrou S , Doyle LW , Draper ES , Marlow N . The cost of preterm birth throughout childhood in England and Wales. Pediatrics. 2009;123(2):e312–27. 19171583 10.1542/peds.2008-1827

[3] Moster D , Lie RT , Markestad T . Long‐term medical and social consequences of preterm birth. N Engl J Med. 2008;359(3):262–73. 18635431 10.1056/NEJMoa0706475

[4] Petrou S , Yiu HH , Kwon J . Economic consequences of preterm birth: a systematic review of the recent literature (2009–2017). Arch Dis Child. 2019;104(5):456–65. 30413489 10.1136/archdischild-2018-315778

[5] Khan K , Petrou S , Dritsaki M , Johnson S , Manktelow B , Draper E , et al. Economic costs associated with moderate and late preterm birth: a prospective population‐based study. BJOG. 2015;122(11):1495–505. 26219352 10.1111/1471-0528.13515

[6] Sharp A , Alfirevic Z . Provision and practice of specialist preterm labour clinics: a UK survey of practice. BJOG. 2014;121(4):417–21. 24314110 10.1111/1471-0528.12512

[7] Morisaki N , Togoobaatar G , Vogel J , Souza J , Hogue CR , Jayaratne K , et al. Risk factors for spontaneous and provider‐initiated preterm delivery in high and low human development index countries: a secondary analysis of the World Health Organization multicountry survey on maternal and newborn health. BJOG. 2014;121(s1):101–9. 10.1111/1471-0528.12631 24641540

[8] NHS England . Saving babies' lives: a care bundle for reducing stillbirth [Internet]. 2016 [cited 2022 Jan 10]. Available from: https://www.england.nhs.uk/wp‐content/uploads/2016/03/saving‐babies‐lives‐car‐bundl.pdf

[9] Lucovnik M , Bregar AT , Steblovnik L , Verdenik I , Gersak K , Blickstein I , et al. Changes in incidence of iatrogenic and spontaneous preterm births over time: a population‐based study. J Perinat Med. 2016;44(5):505–9. 26646020 10.1515/jpm-2015-0271

[10] Chappell LC , Brocklehurst P , Green ME , Hunter R , Hardy P , Juszczak E , et al. Planned early delivery or expectant management for late preterm pre‐eclampsia (PHOENIX): a randomised controlled trial. Lancet. 2019;394(10204):1181–90. 31472930 10.1016/S0140-6736(19)31963-4 PMC6892281

[11] Lisonkova S , Hutcheon JA , Joseph KS . Temporal trends in neonatal outcomes following iatrogenic preterm delivery. BMC Pregnancy Childbirth. 2011;11(1):39. 21612655 10.1186/1471-2393-11-39 PMC3130708

[12] Zeitlin J , Szamotulska K , Drewniak N , Mohangoo A , Chalmers J , Sakkeus L , et al. Preterm birth time trends in Europe: a study of 19 countries. BJOG. 2013;120(11):1356–65. 23700966 10.1111/1471-0528.12281 PMC4285908

[13] van Zijl MD , Koullali B , Oudijk MA , Ravelli ACJ , Mol BWJ , Pajkrt E , et al. Trends in preterm birth in singleton and multiple gestations in the Netherlands 2008–2015: a population‐based study. Eur J Obstet Gynecol Reprod Biol. 2020;247:111–5. 32087421 10.1016/j.ejogrb.2020.02.021

[14] Chen C , Zhang JW , Xia HW , Zhang HX , Betran AP , Zhang L , et al. Preterm birth in China between 2015 and 2016. Am J Public Health. 2019;109(11):1597–604. 31536409 10.2105/AJPH.2019.305287 PMC6775901

[15] Grétarsdóttir ÁS , Aspelund T , Steingrímsdóttir Þ , Bjarnadóttir RI , Einarsdóttir K . Preterm births in Iceland 1997‐2016: preterm birth rates by gestational age groups and type of preterm birth. Birth. 2020;47(1):105–14. 31746027 10.1111/birt.12467

[16] Burger RJ , Temmink JD , Wertaschnigg D , Ganzevoort W , Reddy M , Davey M , et al. Trends in singleton preterm birth in Victoria, 2007 to 2017: a consecutive cross‐sectional study. Acta Obstet Gynecol Scand. 2021;100(7):1230–8. 33382080 10.1111/aogs.14074 PMC8359202

[17] Norman JE , Shennan AH . Prevention of preterm birth—why can't we do any better? Lancet. 2013;381(9862):184–5. 23158880 10.1016/S0140-6736(12)61956-4

[18] Goldenberg RL , Culhane JF , Iams JD , Romero R . Epidemiology and causes of preterm birth. Lancet. 2008;371(9606):75–84. 18177778 10.1016/S0140-6736(08)60074-4 PMC7134569

[19] NMPA Project Team . National Maternity and Perinatal Audit clinical report 2017: revised version. London: Royal College of Obstetricians and Gynaecologists; 2018.

[20] NMPA Project Team . National Maternity and Perinatal Audit: clinical report 2019 [Internet]. 2019 [cited 2020 Nov 2]. Available from: https://maternityaudit.org.uk/FilesUploaded/NMPA%20Clinical%20Report%202019.pdf

[21] Department for Communities and Local Government . The English indices of deprivation 2015 statistical release [Internet]. 2015 [cited 2022 Jan 14]. Available from: https://www.gov.uk/government/statistics/english‐indices‐of‐deprivation‐2015

[22] Cromwell D , Knight H , Gurol‐Urganci I . Parity derived for pregnant women using historical administrative hospital data: accuracy varied among patient groups. J Clin Epidemiol. 2014;67(5):578–85. 24411310 10.1016/j.jclinepi.2013.10.011

[23] Knight H , Gurol‐Urganci I , Meulen J , Mahmood T , Richmond D , Dougall A , et al. Vaginal birth after caesarean section: a cohort study investigating factors associated with its uptake and success. BJOG. 2014;121(2):183–92. 24251861 10.1111/1471-0528.12508

[24] Kirkwood B , Sterne JA . Essential medical statistics. 2nd ed. Hoboken: Blackwell Science; 2003.

[25] White IR , Royston P , Wood AM . Multiple imputation using chained equations: issues and guidance for practice. Stat Med. 2011;30(4):377–99. 21225900 10.1002/sim.4067

[26] Spiegelhalter DJ . Funnel plots for comparing institutional performance. Stat Med. 2005;24(8):1185–202. 15568194 10.1002/sim.1970

[27] Network UPC . Reducing preterm birth: guidelines for Commissioners and Providers [Internet]. Tommy's; 2019 [cited 2022 Sep 27]. Available from: https://www.tommys.org/sites/default/files/2021‐03/reducingpretermbirthguidance19.pdf

[28] Blencowe H , Cousens S , Jassir FB , Say L , Chou D , Mathers C , et al. National, regional, and worldwide estimates of stillbirth rates in 2015, with trends from 2000: a systematic analysis. Lancet Glob Health. 2016;4(2):e98–108. 26795602 10.1016/S2214-109X(15)00275-2

[29] Oliver‐Williams C , Fleming M , Wood A , Smith G . Previous miscarriage and the subsequent risk of preterm birth in Scotland, 1980‐2008: a historical cohort study. BJOG. 2015;122(11):1525–34. 25626593 10.1111/1471-0528.13276 PMC4611958

[30] ONS . Childbearing for women born in different years, England and Wales: 2019 [Internet]. 2020 [cited 2022 Jan 16]. Available from: https://www.ons.gov.uk/peoplepopulationandcommunity/birthsdeathsandmarriages/conceptionandfertilityrates/bulletins/childbearingforwomenbornindifferentyearsenglandandwales/2019

[31] NHS England . The NHS long term plan. 2019.

[32] Soneji S , Beltrán‐Sánchez H . Association of maternal cigarette smoking and smoking cessation with preterm birth. JAMA Netw Open. 2019;2(4):e192514. 31002320 10.1001/jamanetworkopen.2019.2514 PMC6481448

[33] Wagijo M , Sheikh A , Duijts L , Been JV . Reducing tobacco smoking and smoke exposure to prevent preterm birth and its complications. Paediatr Respir Rev. 2017;22:3–10. 26482273 10.1016/j.prrv.2015.09.002

[34] Medicine BA for P . Antenatal optimisation for preterm infants less than 34 weeks: a quality improvement toolkit [Internet]. 2020 [cited 2022 Jan 14]. Available from: https://www.bapm.org/pages/194‐antenatal‐optimisation‐toolkit

[35] Smith GCS , Shah I , Pell JP , Crossley JA , Dobbie R . Maternal obesity in early pregnancy and risk of spontaneous and elective preterm deliveries: a retrospective cohort study. Am J Public Health. 2007;97(1):157–62. 17138924 10.2105/AJPH.2005.074294 PMC1716235

