# Supplementary material for: Iatrogenic and spontaneous preterm birth in England: A population‐based cohort study
Source: BJOG. 2022 Oct 3;130(1):33–41. doi: 10.1111/1471-0528.17291 (PMC10092353; doi:10.1111/1471-0528.17291)
Supplement: Supplementary file 1 — Appendix S1 [file BJO-130-33-s008.docx]

**Supplementary Information for**

**Iatrogenic and spontaneous preterm birth in England: population-based cohort study**

*Supplementary Table 1.* Definition and source of variables used in defining outcomes

*Supplementary Table 2.* Diagnostic codes used to identify maternal medical conditions and possible indications for preterm birth

*Supplementary Table 3.* Number of births by gestation in weeks among 963 800 singleton live births in England in 2015-17

*Supplementary Table 4.* Frequency of recorded codes that may represent indications for preterm birth among 31 097 iatrogenic preterm births recorded in England in 2015 -17 (data for figure 3)

*Supplementary Table 5.* Complete case analysis among 646 193 women who gave birth in England between 1^st^ April 2015 and 31^st^ March 2017 to a singleton live infant and had complete information about all covariates

*Supplementary Table 6.* Summary results of sensitivity analysis which excluded preterm births associated with possible PPROM: results from analysis of 955 099 women who gave birth between 1^st^ April 2015 and 31^st^ March 2017

*Supplementary Table 7.* Results of sensitivity analysis which incorporated adjustment for maternal medical conditions

*Supplementary Figure S1.* Flow chart

*Supplementary Figure 2.* Singleton iatrogenic and spontaneous births at each week of gestation among 963 800 women who gave birth in England between 1^st^ April 2015 and 31^st^ March 2017

**Supplementary Table 1. Definition and source of variables used in defining outcomes**

| **Definition** | **Variables Required** | **Source** | **Values included in measure** |
| --- | --- | --- | --- |
| Preterm birth | Gestational age | MIS^1^ | <37 completed weeks |
|  | AND Fetus outcome | MIS | 10 = live birth |
| Iatrogenic preterm birth | Labour onset | MIS | 3,4,5 = induction of labour; 2= prelabour caesarean section |
|  | OR Delivery method | MIS | 7 = elective caesarean birth |
|  | AND Gestational age | MIS | <37 completed weeks |
|  | AND Fetus outcome | MIS | 10 = live birth |
| Spontaneous preterm birth | Labour onset | MIS | 1 = spontaneous |
|  | AND Delivery method | MIS | Any excluding 7 |
|  | AND Gestational age | MIS | <37 completed weeks |
|  | AND Fetus outcome | MIS | 10 = live birth |
| Iatrogenic preterm birth without coded indication | Diagnosis codes attributed to birth episode | HES^2^ | See Supplementary table 2 |
| Preterm birth associated with prolonged preterm rupture of membranes (PPROM) | Diagnosis codes attributed to birth episode | HES | See Supplementary table 2 |

1. MIS = maternity information system
2. HES = Hospital episode statistics

**Supplementary Table 2. Diagnostic codes used to identify maternal medical conditions and possible indications for preterm birth**

|  | Codes in current episode |
| --- | --- |
| Hypertensive disease |  |
| Pre existing | O10, I10, I11, I12, I13, I15 |
| New onset hypertensive disease in pregnancy | O11, O12, O13, O14, O15, O16 |
| Diabetes |  |
| Pre-existing diabetes | O24.0, E10, E11 |
| Gestational diabetes | O24.1 |
| Unspecified | O24.9 |
| Liver conditions | O26.6 |
| Infection | O98 |
| Fetal malformation | O35 |
| Fetal isoimmunisation | O36.0, O36.1 |
| Fetal growth restriction | O36.5 |
| Oligo or anhydramnios | O41.0 |
| Chorioamnionitis | O36.5 |
| Prolonged preterm rupture of membranes | O42.1 |
| Placental conditions | O43,O44,O45,O46,O69.4 |
| Maternal cardiac disease | O99.4 |
| Previous poor obstetric outcome | Z35.2 |
| Cervical abnormality | O34.3, O34.4, O71.3, Q51.1 |
| Group B streptococcus | B95.1 |
| Urinary tract infection | N39.0, O23 |
| Antepartum haemorrhage | O46 |
| Abruption | O45 |
| Partial abortion of second twin | O31.1,O31.3 |
| Fetal distress | O68 |

**Supplementary Table 3. Number of births by gestation in weeks among 963 800 singleton live births in England in 2015-17**

| **Gestation in weeks** | **Overall number of births** | | **Iatrogenic** | | **Spontaneous** | |
| --- | --- | --- | --- | --- | --- | --- |
|  | n | % | n | % | n | % |
|  | 963 800 |  | 417 689 |  | 546 111 |  |
| 22^+0^–22^+6^ | 151 | 0.02% | 31 | 0.01% | 120 | 0.02% |
| 23^+0^-23^+6^ | 313 | 0.03% | 47 | 0.01% | 266 | 0.05% |
| 24^+0^-24^+6^ | 512 | 0.05% | 102 | 0.02% | 410 | 0.08% |
| 25^+0^-25^+6^ | 569 | 0.06% | 168 | 0.04% | 401 | 0.07% |
| 26^+0^-26^+6^ | 661 | 0.07% | 253 | 0.06% | 408 | 0.07% |
| 27^+0^-27^+6^ | 817 | 0.08% | 375 | 0.09% | 442 | 0.08% |
| 28^+0^-28^+6^ | 1088 | 0.11% | 601 | 0.14% | 487 | 0.09% |
| 29^+0^-29^+6^ | 1156 | 0.12% | 619 | 0.15% | 537 | 0.10% |
| 30^+0^-30^+6^ | 1510 | 0.16% | 811 | 0.19% | 699 | 0.13% |
| 31^+0^-31^+6^ | 2045 | 0.21% | 1038 | 0.25% | 1007 | 0.18% |
| 32^+0^-32^+6^ | 2667 | 0.28% | 1385 | 0.33% | 1282 | 0.23% |
| 33^+0^-33^+6^ | 4011 | 0.42% | 1965 | 0.47% | 2046 | 0.37% |
| 34^+0^-34^+6^ | 7448 | 0.77% | 4117 | 0.99% | 3331 | 0.61% |
| 35^+0^-35^+6^ | 11457 | 1.19% | 5909 | 1.41% | 5548 | 1.02% |
| 36^+0^-36^+6^ | 24445 | 2.54% | 13676 | 3.27% | 10769 | 1.97% |
| 37^+0^-37^+6^ | 67380 | 6.99% | 42407 | 10.15% | 24973 | 4.57% |
| 38^+0^-38^+6^ | 137210 | 14.24% | 75804 | 18.15% | 61406 | 11.24% |
| 39^+0^-39^+6^ | 249279 | 25.86% | 115493 | 27.65% | 133786 | 24.50% |
| 40^+0^-40^+6^ | 260912 | 27.07% | 70048 | 16.77% | 190864 | 34.95% |
| 41^+0^-41^+6^ | 166027 | 17.23% | 64357 | 15.41% | 101670 | 18.62% |
| 42^+0^-42^+6^ | 24142 | 2.50% | 18483 | 4.43% | 5659 | 1.04% |

**Supplementary Table 4. Frequency of recorded codes that may represent indications for preterm birth among 31 097 iatrogenic preterm births recorded in England in 2015 -17 (data for figure 3)**

| **Indication** | All iatrogenic preterm births | | Gestation <28 weeks | | Gestation 28-31 weeks | | Gestation 32-36 weeks | |
| --- | --- | --- | --- | --- | --- | --- | --- | --- |
|  | n | % | n | % | n | % | n | % |
| *Total* | 31097 |  | 976 |  | 6419 |  | 23702 |  |
| Hypertensive disease | 5599 | 18.0% | 231 | 23.7% | 1794 | 27.9% | 3574 | 15.1% |
| Diabetes | 4196 | 13.5% | 231 | 23.7% | 670 | 10.4% | 3483 | 14.7% |
| Liver conditions | 843 | 2.7% | *#* | *#* | 76 | 1.2% | 763 | 3.2% |
| Infection | 277 | 0.9% | 17 | 1.7% | 71 | 1.1% | 189 | 0.8% |
| Fetal malformation | 702 | 2.3% | 31 | 3.2% | 165 | 2.6% | 506 | 2.1% |
| Fetal isoimmunisation | 228 | 0.7% | *#* | *#* | 33 | 0.5% | 192 | 0.8% |
| Fetal growth restriction | 7241 | 23.3% | 211 | 21.6% | 1760 | 27.4% | 5270 | 22.2% |
| Oligo or anhydramnios | 1666 | 5.4% | 76 | 7.8% | 422 | 6.6% | 1168 | 4.9% |
| Chorioamnionitis | 535 | 1.7% | 129 | 13.2% | 256 | 4.0% | 150 | 0.6% |
| Prolonged preterm rupture of membranes | 3421 | 11.0% | 97 | 9.9% | 423 | 6.6% | 2901 | 12.2% |
| Placental conditions | 4434 | 14.3% | 277 | 28.4% | 1430 | 22.3% | 2727 | 11.5% |
| Maternal cardiac disease | 245 | 0.8% | 12 | 1.2% | 65 | 1.0% | 168 | 0.7% |
| Previous poor obstetric outcome | 1919 | 6.2% | 78 | 8.0% | 365 | 5.7% | 1476 | 6.2% |
| Cervical abnormality | 281 | 0.9% | 25 | 2.6% | 67 | 1.0% | 189 | 0.8% |
| Group B streptococcus | 118 | 0.4% | 5 | 0.5% | 28 | 0.4% | 85 | 0.4% |
| Urinary tract infection | 553 | 1.8% | 23 | 2.4% | 136 | 2.1% | 394 | 1.7% |
| Antepartum haemorrhage | 1502 | 4.8% | 100 | 10.2% | 445 | 6.9% | 957 | 4.0% |
| Abruption | 1041 | 3.3% | 108 | 11.1% | 434 | 6.8% | 499 | 2.1% |
| Partial abortion of second twin | 28 | 0.1% | *#* | *#* | 14 | 0.2% | 11 | 0.0% |
| Fetal distress | 7906 | 25.4% | 211 | 21.6% | 1909 | 29.7% | 5786 | 24.4% |

*# small numbers are suppressed to prevent identification*

**Supplementary Table 5. Complete case analysis among 646 193 women who gave birth in England between 1^st^ April 2015 and 31^st^ March 2017 to a singleton live infant and had complete information about all covariates**

| Maternal characteristics | | Spontaneous preterm birth (n= 17 938) | | | Iatrogenic preterm birth (n= 20 790) | | |
| --- | --- | --- | --- | --- | --- | --- | --- |
|  |  | Crude rate ratio (95% CI)* | Adjusted rate ratio (95% CI)† | p | Crude rate ratio (95% CI)* | Adjusted rate ratio (95% CI)† | p-value |
| **Maternal age** | <20 | 1.63 (1.54, 1.72) | 1.29 (1.20, 1.38) | <0.001 | 1.23 (1.15, 1.30) | 1.17 (1.08, 1.26) | <0.001 |
|  | 20-24 | 1.16 (1.12, 1.20) | 1.05 (1.01, 1.10) |  | 1.12 (1.08, 1.16) | 1.08 (1.03, 1.13) |  |
|  | 25-29 | Ref | Ref |  | Ref | Ref |  |
|  | 30-34 | 0.98 (0.95, 1.01) | 1.03 (1.00, 1.07) |  | 0.98 (0.95, 1.01) | 1.25 (1.20, 1.30) |  |
|  | 35-39 | 0.97 (0.93, 1.00) | 1.10 (1.05, 1.15) |  | 1.16 (1.13, 1.20) | 1.41 (1.34, 1.48) |  |
|  | 40+ | 1.01 (0.95, 1.08) | 1.17 (1.09, 1.27) |  | 1.74 (1.66, 1.82) | 1.58 (1.48, 1.68) |  |
|  |  |  |  |  |  |  |  |
| **Maternal BMI** | <18.5 | 1.66 (1.57, 1.76) | 1.41 (1.32, 1.51) | <0.001 | 1.36 (1.27, 1.45) | 1.26 (1.17, 1.35) | <0.001 |
|  | 18.5-24.9 | Ref | Ref |  | Ref | Ref |  |
|  | 25-29.9 | 0.89 (0.86, 0.91) | 0.87 (0.84, 0.90) |  | 1.17 (1.13, 1.20) | 1.09 (1.06, 1.13) |  |
|  | 30-34.9 | 0.84 (0.80, 0.87) | 0.80 (0.77, 0.84) |  | 1.41 (1.36, 1.46) | 1.25 (1.20, 1.30) |  |
|  | 35-39.9 | 0.80 (0.75, 0.85) | 0.76 (0.71, 0.82) |  | 1.62 (1.55, 1.70) | 1.41 (1.34, 1.48) |  |
|  | 40+ | 0.76 (0.69, 0.83) | 0.71 (0.64, 0.78) |  | 1.90 (1.79, 2.01) | 1.58 (1.48, 1.68) |  |
|  |  |  |  |  |  |  |  |
| **Ethnicity** | White | Ref | Ref | <0.001 | Ref | Ref | <0.001 |
|  | S. Asian | 1.06 (1.02, 1.10) | 1.11 (1.06, 1.16) |  | 1.09 (1.05, 1.13) | 1.06 (1.02, 1.10) |  |
|  | Black | 0.93 (0.87, 0.98) | 0.97 (0.90, 1.05) |  | 1.26 (1.20, 1.33) | 1.10 (1.04, 1.17) |  |
|  | Mixed | 1.02 (0.93, 1.12) | 1.01 (0.91, 1.13) |  | 1.10 (1.01, 1.19) | 1.09 (0.99, 1.20) |  |
|  | Other | 1.00 (0.95, 1.07) | 1.11 (1.03, 1.19) |  | 0.79 (0.75, 0.84) | 0.84 (0.78, 0.91) |  |
|  |  |  |  |  |  |  |  |
| **IMD (1= least deprived; 5= most deprived)** | 1 | Ref | Ref | <0.001 | Ref | Ref | <0.001 |
|  | 2 | 1.07 (1.02, 1.12) | 1.06 (1.01, 1.13) |  | 1.06 (1.01, 1.11) | 1.02 (0.97, 1.08) |  |
|  | 3 | 1.11 (1.06, 1.16) | 1.08 (1.02, 1.13) |  | 1.14 (1.09, 1.19) | 1.08 (1.03, 1.14) |  |
|  | 4 | 1.21 (1.16, 1.26) | 1.12 (1.07, 1.18) |  | 1.28 (1.23, 1.33) | 1.16 (1.10, 1.21) |  |
|  | 5 | 1.38 (1.33, 1.44) | 1.24 (1.18, 1.30) |  | 1.49 (1.44, 1.54) | 1.27 (1.21, 1.33) |  |
|  | |  |  |  |  |  |  |
| **Smoking at booking** | | 1.80 (1.75, 1.86) | 1.60 (1.54, 1.66) | <0.001 | 1.67 (1.62, 1.72) | 1.50 (1.45, 1.55) | <0.001 |
|  |  |  |  |  |  |  |  |
| **Parity** | 0 | Ref | Ref | <0.001 | Ref | Ref | <0.001 |
|  | 1 | 0.77 (0.75, 0.80) | 0.63 (0.61, 0.66) |  | 0.90 (0.87, 0.92) | 0.63 (0.61, 0.66) |  |
|  | 2 | 0.87 (0.84, 0.91) | 0.61 (0.58, 0.64) |  | 1.17 (1.13, 1.21) | 0.72 (0.69, 0.75) |  |
|  | 3+ | 1.14 (1.10, 1.19) | 0.63 (0.60, 0.67) |  | 1.70 (1.64, 1.75) | 0.85 (0.81, 0.90) |  |
|  |  |  |  |  |  |  |  |
| **Previous caesarean section** | | 0.93 (0.90, 0.96) | 0.85 (0.82, 0.89) | <0.001 | 1.96 (1.91, 2.01) | 1.89 (1.83, 1.96) | <0.001 |
|  |  |  |  |  |  |  |  |
| **Previous preterm birth** | | 5.27 (5.12, 5.42) | 6.81 (6.55, 7.09) | <0.001 | 3.77 (3.66, 3.89) | 3.27 (3.14, 3.40) | <0.001 |
| *rate ratio compared to term births. †compared to term births, adjusted for listed factors | | | | | | | |

**Supplementary Table 6. Summary results of sensitivity analysis which excluded preterm births associated with possible PPROM: results from analysis of 955 099 women who gave birth between 1^st^ April 2015 and 31^st^ March 2017**

| Maternal characteristics | | Spontaneous preterm birth (n= 24 370) | | | Iatrogenic preterm birth (n= 25 779) | | |
| --- | --- | --- | --- | --- | --- | --- | --- |
|  |  | Crude rate ratio (95% CI)* | Adjusted rate ratio (95% CI)† | p | Crude rate ratio (95% CI)* | Adjusted rate ratio (95% CI)† | p-value |
| **Maternal age** | <20 | 1.21 (1.13, 1.29) | 1.35 (1.27, 1.44) | <0.001 | 1.67 (1.57, 1.77) | 1.11 (1.03, 1.19) | <0.001 |
|  | 20-24 | 1.10 (1.06, 1.14) | 1.06 (1.02, 1.10) |  | 1.17 (1.12, 1.21) | 1.06 (1.02, 1.10) |  |
|  | 25-29 | Ref | Ref |  | Ref | Ref |  |
|  | 30-34 | 1.00 (0.96, 1.03) | 1.04 (1.00, 1.07) |  | 0.97 (0.94, 1.00) | 1.03 (1.00, 1.07) |  |
|  | 35-39 | 1.21 (1.16, 1.25) | 1.09 (1.05, 1.13) |  | 0.97 (0.93, 1.01) | 1.23 (1.19, 1.28) |  |
|  | 40+ | 1.86 (1.77, 1.96) | 1.17 (1.09, 1.25) |  | 1.01 (0.94, 1.08) | 1.81 (1.72, 1.91) |  |
|  |  |  |  |  |  |  |  |
| **Maternal BMI** | <18.5 | 1.37 (1.27, 1.48) | 1.46 (1.37, 1.55) | <0.001 | 1.69 (1.59, 1.80) | 1.28 (1.19, 1.39) | <0.001 |
|  | 18.5-24.9 | Ref | Ref |  | Ref | Ref |  |
|  | 25-29.9 | 1.18 (1.14, 1.22) | 0.90 (0.87, 0.93) |  | 0.89 (0.86, 0.92) | 1.11 (1.08, 1.15) |  |
|  | 30-34.9 | 1.43 (1.38, 1.49) | 0.83 (0.79, 0.87) |  | 0.84 (0.80, 0.88) | 1.28 (1.23, 1.33) |  |
|  | 35-39.9 | 1.67 (1.59, 1.76) | 0.79 (0.74, 0.85) |  | 0.81 (0.76, 0.87) | 1.44 (1.36, 1.51) |  |
|  | 40+ | 2.00 (1.88, 2.13) | 0.75 (0.68, 0.83) |  | 0.78 (0.70, 0.85) | 1.65 (1.55, 1.76) |  |
|  |  |  |  |  |  |  |  |
| **Ethnicity** | White | Ref | Ref | 0.02 | Ref | Ref | <0.001 |
|  | S. Asian | 1.09 (1.05, 1.13) | 1.06 (1.02, 1.10) |  | 1.03 (0.99, 1.07) | 1.06 (1.02, 1.10) |  |
|  | Black | 1.33 (1.26, 1.40) | 0.98 (0.92, 1.05) |  | 0.91 (0.85, 0.97) | 1.13 (1.07, 1.19) |  |
|  | Mixed | 1.09 (1.00, 1.19) | 0.99 (0.89, 1.10) |  | 1.01 (0.91, 1.13) | 1.04 (0.95, 1.14) |  |
|  | Other | 0.78 (0.73, 0.84) | 1.06 (0.99, 1.13) |  | 0.97 (0.91, 1.04) | 0.83 (0.77, 0.89) |  |
|  |  |  |  |  |  |  |  |
| **IMD (1= least deprived; 5= most deprived)** | 1 | Ref | Ref | <0.001 | Ref | Ref | <0.001 |
|  | 2 | 1.06 (1.01, 1.12) | 1.03 (0.98, 1.09) |  | 1.06 (1.01, 1.11) | 1.04 (0.99, 1.10) |  |
|  | 3 | 1.15 (1.10, 1.21) | 1.04 (0.99, 1.09) |  | 1.08 (1.03, 1.13) | 1.10 (1.05, 1.15) |  |
|  | 4 | 1.28 (1.23, 1.34) | 1.09 (1.04, 1.14) |  | 1.19 (1.14, 1.24) | 1.17 (1.12, 1.22) |  |
|  | 5 | 1.50 (1.44, 1.56) | 1.19 (1.14, 1.24) |  | 1.35 (1.30, 1.41) | 1.27 (1.22, 1.33) |  |
|  | |  |  |  |  |  |  |
| **Smoking at booking** | | 1.66 (1.60, 1.71) | 1.59 (1.54, 1.65) | <0.001 | 1.77 (1.71, 1.83) | 1.54 (1.49, 1.60) | <0.001 |
|  |  |  |  |  |  |  |  |
| **Parity** | 0 | Ref | Ref | <0.001 | Ref | Ref | <0.001 |
|  | 1 | 0.90 (0.87, 0.92) | 0.63 (0.61, 0.65) |  | 0.78 (0.76, 0.81) | 0.60 (0.58, 0.62) |  |
|  | 2 | 1.19 (1.15, 1.24) | 0.61 (0.58, 0.63) |  | 0.88 (0.85, 0.92) | 0.67 (0.65, 0.70) |  |
|  | 3+ | 1.73 (1.67, 1.80) | 0.62 (0.59, 0.65) |  | 1.13 (1.08, 1.18) | 0.79 (0.75, 0.82) |  |
|  |  |  |  |  |  |  |  |
| **Previous caesarean section** | | 2.16 (2.10, 2.22) | 0.87 (0.84, 0.91) | <0.001 | 0.94 (0.91, 0.98) | 2.08 (2.01, 2.15) | <0.001 |
|  |  |  |  |  |  |  |  |
| **Previous preterm birth** | | 3.93 (3.80, 4.06) | 6.83 (6.59, 7.08) | <0.001 | 5.48 (5.32, 5.65) | 3.38 (3.26, 3.50) | <0.001 |
| *rate ratio compared to term births. †compared to term births, adjusted for listed factors | | | | | | | |

**Supplementary Table 7. Results of sensitivity analysis which incorporated adjustment for maternal medical conditions**

| Characteristic | | Spontaneous preterm (n=27 753) | | | | Iatrogenic preterm (n=31 097) | | | |
| --- | --- | --- | --- | --- | --- | --- | --- | --- | --- |
|  |  | Rate | Crude rate ratio (95% CI)* | Adjusted rate ratio (95% CI)† | p | Rate | Crude rate ratio (95% CI)* | Adjusted rate ratio (95% CI)† | p-value |
| **Maternal age** | <20 | 4.55 | 1.63 (1.54, 1.72) | 1.22 (1.13, 1.32) | <0.001 | 3.67 | 1.23 (1.15, 1.30) | 1.13 (1.06, 1.21) | <0.001 |
|  | 20-24 | 3.23 | 1.16 (1.12, 1.20) | 0.94 (0.88, 1.00) |  | 3.36 | 1.12 (1.08, 1.16) | 1.08 (1.04, 1.12) |  |
|  | 25-30 | 2.79 | Ref | Ref |  | 2.99 | Ref | Ref |  |
|  | 30-34 | 2.73 | 0.98 (0.95, 1.01) | 1.09 (1.03, 1.15) |  | 2.93 | 0.98 (0.95, 1.01) | 0.99 (0.95, 1.02) |  |
|  | 35-39 | 2.70 | 0.97 (0.93, 1.00) | 1.09 (1.01, 1.17) |  | 3.48 | 1.16 (1.13, 1.20) | 1.09 (1.05, 1.13) |  |
|  | 40+ | 2.83 | 1.01 (0.95, 1.08) | 0.90 (0.78, 1.05) |  | 5.20 | 1.74 (1.66, 1.82) | 1.45 (1.38, 1.53) |  |
|  |  |  |  |  |  |  |  |  |  |
| **Maternal BMI** | <18.5 | 4.83 | 1.66 (1.56, 1.76) | 1.43 (1.34, 1.52) | <0.001 | 3.61 | 1.37 (1.28, 1.47) | 1.28 (1.20, 1.38) | <0.001 |
|  | 18.5-24.9 | 2.91 | Ref | Ref |  | 2.66 | Ref | Ref |  |
|  | 25-29.9 | 2.58 | 0.89 (0.86, 0.92) | 0.88 (0.85, 0.91) |  | 3.10 | 1.16 (1.12, 1.19) | 1.03 (1.00, 1.06) |  |
|  | 30-34.9 | 2.43 | 0.84 (0.81, 0.88) | 0.81 (0.77, 0.85) |  | 3.74 | 1.39 (1.34, 1.44) | 1.09 (1.04, 1.13) |  |
|  | 35-39.9 | 2.33 | 0.82 (0.77, 0.87) | 0.78 (0.72, 0.84) |  | 4.31 | 1.60 (1.53, 1.68) | 1.12 (1.07, 1.19) |  |
|  | 40+ | 2.21 | 0.79 (0.72, 0.86) | 0.72 (0.65, 0.80) |  | 5.04 | 1.87 (1.76, 1.98) | 1.13 (1.05, 1.21) |  |
|  |  |  |  |  |  |  |  |  |  |
| **Ethnicity** | White | 2.86 | Ref | Ref | 0.002 | 3.22 | Ref | Ref | <0.001 |
|  | S. Asian | 3.02 | 1.04 (1.01, 1.08) | 1.09 (1.04, 1.14) |  | 3.52 | 1.10 (1.06, 1.13) | 0.97 (0.94, 1.01) |  |
|  | Black | 2.65 | 0.92 (0.87, 0.97) | 0.98 (0.91, 1.05) |  | 4.06 | 1.27 (1.21, 1.33) | 0.98 (0.92, 1.03) |  |
|  | Mixed | 2.93 | 1.03 (0.93, 1.13) | 1.01 (0.90, 1.13) |  | 3.53 | 1.10 (1.01, 1.19) | 1.08 (0.99, 1.18) |  |
|  | Other | 2.87 | 0.99 (0.93, 1.05) | 1.08 (1.01, 1.16) |  | 2.55 | 0.80 (0.75, 0.85) | 0.82 (0.76, 0.87) |  |
|  |  |  |  |  |  |  |  |  |  |
| **IMD (1= least deprived; 5= most deprived)** | 1 | 2.41 | Ref | Ref | <0.001 | 2.62 | Ref | Ref | <0.001 |
|  | 2 | 2.58 | 1.07 (1.02, 1.12) | 1.06 (1.00, 1.12) |  | 2.77 | 1.05 (1.01, 1.10) | 1.03 (0.97, 1.08) |  |
|  | 3 | 2.68 | 1.11 (1.06, 1.16) | 1.06 (1.01, 1.12) |  | 2.99 | 1.14 (1.09, 1.19) | 1.06 (1.01, 1.11) |  |
|  | 4 | 2.92 | 1.21 (1.16, 1.26) | 1.10 (1.05, 1.16) |  | 3.35 | 1.27 (1.22, 1.32) | 1.11 (1.07, 1.16) |  |
|  | 5 | 3.34 | 1.38 (1.33, 1.44) | 1.21 (1.44, 1.27) |  | 3.90 | 1.48 (1.43, 1.54) | 1.21 (1.16, 1.26) |  |
|  | |  |  |  |  |  |  |  |  |
| **Smoking status at booking** | non-smoker | 2.52 | Ref | Ref |  | 2.92 | Ref | Ref | <0.001 |
|  | smoker | 4.55 | 1.78 (1.73, 1.84) | 1.67 (1.58, 1.76) | <0.001 | 4.88 | 1.68 (1.63, 1.73) | 1.59 (1.54, 1.65) |  |
|  |  |  |  |  |  |  |  |  |  |
| **Parity** | 0 | 3.15 | Ref | Ref | <0.001 | 3.09 | Ref | Ref | <0.001 |
|  | 1 | 2.44 | 0.77 (0.75, 0.80) | 0.53 (0.35, 0.80) |  | 2.76 | 0.89 (0.87, 0.92) | 0.73 (0.70, 0.75) |  |
|  | 2 | 2.75 | 0.87 (0.84, 0.91) | 0.49 (0.37, 0.64) |  | 3.61 | 1.17 (1.13, 1.21) | 0.85 (0.82, 0.89) |  |
|  | 3+ | 3.59 | 1.14 (1.10, 1.19) | 0.83 (0.68, 1.01) |  | 5.24 | 1.70 (1.64, 1.75) | 1.02 (0.98, 1.07) |  |
|  |  |  |  |  |  |  |  |  |  |
| **Previous caesarean section** | | 2.70 | 0.93 (0.90, 0.96) | 0.86 (0.82, 0.90) | <0.001 | 5.57 | 1.97 (1.92, 2.02) | 1.74 (1.69, 1.80) | <0.001 |
|  |  |  |  |  |  |  |  |  |  |
| **Previous preterm birth** | | 12.8 | 5.27 (5.12, 5.42) | 6.73 (6.47, 7.00) | <0.001 | 10.9 | 3.77 (3.66, 3.99) | 2.90 (2.80, 3.01) | <0.001 |
|  | |  |  |  |  |  |  |  |  |
| **Comorbidity** | |  |  |  |  |  |  |  |  |
| Pre-existing hypertension | | 2.70 | 0.94 (0.80, 1.10) | 0.88 (0.73, 1.06) | 0.09 | 13.02 | 4.10 (3.83, 4.40) | 2.77 (2.54, 3.00) | <0.001 |
| Diabetes (previous or gestational) | | 3.52 | 1.24 (1.19, 1.30) | 1.25 (1.14, 1.38) | <0.001 | 7.38 | 2.49 (2.42, 2.57) | 2.16 (2.01, 2.31) | <0.001 |
| Pre-eclampsia or eclampsia | | 2.09 | 0.72 (0.65, 0.80) | 0.68 (0.56, 0.72) | <0.001 | 25.73 | 9.19 (8.94, 9.45) | 8.34 (8.07, 8.62) | <0.001 |
| *rate ratio compared to term births. †compared to term births, adjusted for listed factors and interaction between BMI and diabetes | | | | | | | | | |

**Supplementary Figure S1. Flow Chart**

Linked maternity dataset for England 2015/17

1 254 484 births in 133 trusts

Births in trusts meeting quality checks

1 035 718 births in 110 trusts

**Trust-level exclusions**

Births in trusts with poor quality data

n=183 112 births (19 trusts)

Births in trusts where data cannot be linked to HES

n=35 654 births (4 trusts)

**Record level exclusions, n=71 918**

Non-singleton births*: n=40 329

Gestation length <22 weeks: n=116

Gestation length >42 weeks: n=2000

Gestation length missing: n=5 918

Stillbirth, termination or miscarriage: n=11 543

Delivery method missing: n=2 729

Labour onset missing: n=9 283

*either not recorded or multiple birth

Singleton live births meeting quality checks

963 800 births in 110 trusts

**Supplementary Figure S2. Singleton iatrogenic and spontaneous births at each week of gestation among 963 800 women who gave birth in England between 1^st^ April 2015 and 31^st^ March 2017**
